# Impact of viral load kinetics and recurrent cytomegalovirus infection in kidney transplantation

**DOI:** 10.3389/fimmu.2025.1600289

**Published:** 2025-08-08

**Authors:** Sabina Dobrer, Karen R. Sherwood, Kimberly Davis, James H. Lan, John Gill, Nancy Matic, Paul A. Keown

**Affiliations:** ^1^ Department of Pathology and Laboratory Medicine, University of British Columbia, Vancouver, BC, Canada; ^2^ Global Evidence & Outcomes Within R&D, Takeda Development Center Americas, Inc., Lexington, MA, United States; ^3^ Department of Medicine, University of British Columbia, Vancouver, BC, Canada

**Keywords:** kidney transplant, cytomegalovirus, CMV, recurrent infection, viral load kinetics, clinical outcomes

## Abstract

**Background:**

We have shown that viral load kinetics during the first cytomegalovirus (CMV) viremic episode are important predictors of kidney transplant failure. This article evaluates the incremental hazard of recurrent CMV viremia and of viral load kinetics on graft and patient survival.

**Methods:**

This retrospective cohort study included 2,464 sequential kidney transplants performed between 2008 and 2018. Care was delivered according to a uniform provincial protocol, and patients were followed for up to 13 years with standardized therapy and continuous monitoring of clinical course, CMV infection, viral load kinetics, and graft and patient outcomes.

**Results:**

434/2,464 (17.6%) patients (age range: 2–80 years) developed CMV infection, of whom 67/434 (15.4%) had 150 episodes of recurrent infection. Mean cumulative CMV frequency reached an asymptote of 21% at 500 days, with the highest rate (43%) in D+/R-, and lowest (1%) in D-/R- risk groups. Multinomial adjusted regression described a composite risk phenotype that included increased age, non-Caucasian race, diabetes, D+/R- status, and delayed graft function (*p*<0.005). Median cumulative viral load kinetic values rose progressively with the number of viremic episodes, maximum viral load rising from 3.8–5.1 log_10_ IU/mL, mean duration of viremia from 15–116 days, and viral AUC from 56.1–492.9 log_10_ IU/mL*days in patients with multiple episodes of CMV viremia. Predicted probability of graft failure and death were closely related to the cumulative duration of viremia and total viral load, with respective survival values declining to 30% and 7% in patients with elevated viremic indices and defined composite risk phenotype.

**Conclusions:**

Patients with a recurrent CMV viremia post-transplant are at exceptionally high risk of transplant failure as measured by graft loss or death, which is determined by both composite risk phenotype and CMV viral load kinetics. Conventional prophylaxis appears to be inadequate to protect these patients from recurrent infection and its serious consequences, and alternative treatment strategies, with continuous long-term monitoring and rapid, effective therapy, are vital to maximize transplant success.

## 1 Introduction

The Genome Canada Transplant Consortium links universities across Canada, the United States, and the European Union to apply precision medicine principles to prevent transplant failure. Post-transplant viral infection remains an important risk, and cytomegalovirus (CMV) is a major therapeutic challenge (1–5). Defined risk modifiers include the pre-transplant serological status of the donor and recipient, the immunosuppressive therapy employed, and the use of viral prophylaxis (3, 6, 7). Specific recommendations for prevention and treatment of CMV infection have been established (1, 2), which include antiviral prophylaxis and preemptive therapy (3, 6). However, current antiviral agents entail important toxicities and are only partially successful in preventing CMV infection (8). CMV viremia or clinical infection is often simply delayed, and recurrent or refractory infection may occur with tissue invasion, superinfection, breakthrough rejection, and graft loss, altering the economic costs and benefit of transplantation.

Our prior report of a large longitudinal Canadian cohort (9) demonstrated that viral load kinetic parameters of a first viremic episode correlate closely with CMV severity and graft loss and provide an index of risk that may be valuable in guiding treatment to prevent transplant failure. Maximum viral load, the duration of viremia, and the viral load area under the curve (AUC) were significantly increased in patients with more severe clinical disease or with graft loss (*p*=0.001). A first CMV viremic episode >15 days or maximum viral load ≥4.0 log_10_ IU/mL predicted a 3-fold increase in the risk of transplant failure (9). Here, we extend this analysis to investigate the incremental hazard of recurrent CMV viremia and of viral load kinetics for first and recurrent episodes on graft and patient survival.

## 2 Methods

### 2.1 Study design and data sources

A retrospective, longitudinal cohort design was employed to examine the probabilities, risk factors, treatments, and outcomes of CMV infection following kidney transplantation. The study cohort and methods are described in detail in our prior study and are summarized here for completeness (9). Transplantation was performed within a provincial renal care program with continuous medical follow-up. Pediatric and adult patients transplanted between January 1, 2008, and December 31, 2018, were selected to ensure standardized diagnostic and therapeutic practices, and were followed from the day of transplantation until December 31, 2019, providing a minimum observation of 1 year and a maximum of 12 years (over 2 million days) of continuous medical supervision after transplantation. Clinical, laboratory, therapeutic, and outcomes data were recorded in the British Columbia (BC) Provincial Kidney Transplant Registry, and supplementary information was obtained as required from additional data systems, including the BC Immunology Laboratory, the Renal Transplant Pathology Program, and other sources. The study was reviewed and approved by the institutional clinical research ethics boards of the University of British Columbia and Vancouver Coastal Health, who waived requirement for individual patient consent.

### 2.2 Patient management

Patient evaluation, donor organ allocation, and all diagnostic, procedural, and therapeutic initiatives were performed according to provincial treatment guidelines, reviewed annually by the BC Transplant Management Committee. Patients considered at low immunological risk included those who were receiving a first graft from a normal criteria donor, with a calculated panel reactive antibody <80%, and who did not have donor-specific antibodies on solid-phase assay. These patients received basiliximab, tacrolimus, mycophenolate mofetil, and rapid prednisone elimination. Those at higher risk routinely received antithymocyte globulin (ATG) for induction therapy with tacrolimus, mycophenolate mofetil, and long-term prednisone treatment. Immune suppression was adjusted by the transplant team for each patient according to time post-transplant, clinical status, and therapeutic concentrations of individual drugs according to a standard provincial management protocol. Graft biopsy was performed for cause and reported by a central team of expert kidney transplant pathologists.

### 2.3 CMV testing

CMV serological status at the time of transplantation was determined by the presence or absence of CMV immunoglobulin G antibodies, and 4 CMV risk groups were defined and categorized as donor (D)-/recipient (R)-, D-/R+, D+/R-, and D+/R+. Measurement of CMV viral load was performed by St. Paul’s Hospital Virology Laboratory using quantitative polymerase chain reaction methodology as previously described (9). Recipient testing was performed pre- and post-transplant as part of routine bloodwork taken approximately weekly for the first 4 weeks, then every 2 weeks to Month 3, every 1 to 2 months to Month 12, and as required after the first post-transplant year. CMV testing was intensified for 6 months following prophylaxis or treatment of infection independent of the time in the transplantation course. A CMV DNA viral load of ≥1,000 copies/mL or ≥830 IU/mL was the threshold for diagnosis of clinically important viremia and commencement of treatment (10–12) in the BC transplant program. The duration of CMV viremia was defined as the time in days from the first to the last positive CMV test for the episode.

### 2.4 CMV prophylaxis and treatment

Adult and pediatric patients who were CMV seronegative and who received a graft from a seropositive donor (D+/R-), CMV-positive pediatric patients, and patients who received ATG induction therapy were treated with valganciclovir prophylaxis for 3 to 6 months. Patients who developed CMV viremia above the treatment threshold but were not on prophylaxis received preemptive valganciclovir treatment for at least 3 weeks until the viremia resolved. Treatment was administered at a dose of 900 mg orally twice daily, or 5 mg/kg intravenously twice daily, adjusted for kidney function and leukopenia. CMV monitoring was performed weekly during the episode of viremia and repeated monthly for at least 2 months after an episode of infection or termination of prophylaxis. Specific medications, doses, and duration of each therapy were obtained from the BC Provincial Transplant database based on pharmacy dispensing data. Treatment episodes were considered separate when the time between courses of therapy was >7 days. CMV-related outcomes were defined based on the Guidelines of the American Society of Transplantation Infectious Disease Community of Practice 2019 (1) adjusted to reflect the longitudinal data available in the EMR. Since CMV end-organ disease was difficult to classify in an observational retrospective study, given the many potential and concomitant causes for gastrointestinal, hepatic, pulmonary, and other dysfunction, this outcome was included in the CMV clinical syndrome.

## 3 Statistical methods

### 3.1 Descriptive statistics

Data review was performed using visualization, tabulation, and other requisite computational processes; missing data were noted but were not imputed for this analysis, and all data discrepancies were reviewed and approved by the research team. Continuous variables were summarized using the number of non-missing observations, mean, standard deviation (SD), median, minimum, and maximum values. Categorical variables were summarized using the number and percentage of patients belonging to each category. The relationships between CMV infection and patient and graft outcomes were determined independently. Pearson’s Chi-squared test (χ²) was used for comparison of categorical variables by different stratifications. For continuous variables, a non-parametric 2-sided Mann–Whitney U-test was used for 2 strata (e.g., living/diseased donor, number of previous transplants) and a non-parametric Kruskal–Wallis H-test for 3 or more categories (e.g., serological donor/patient status).

### 3.2 Regression modeling

Multivariable multinomial regression models were developed to adjust for the influence of covariates on principal outcomes of CMV disease, and graft and patient survival. These included: baseline recipient variables (primary disease diagnosis, age at transplantation, sex, race, number of prior transplants); donor/recipient CMV variables (donor and recipient CMV serological mismatch); donor/transplant variables (type of donor, donor age, sex, race); induction immunosuppression (use of ATG or other biologics); and delayed graft function. The exploratory model approach incorporated both unadjusted and adjusted multinomial logistic regression to examine the relationship between multiple CMV infections and relevant independent variables. The assessment of the proportional odds assumption for the multinomial logistic regression model involved visualization through effect plots, revealing no discernible issues. The analysis aimed to estimate odds ratios (ORs) and their corresponding 95% confidence intervals (CIs) for each category of the outcome variable, using the category of *no CMV infection* during the study period as the reference. Co-linearity of included covariates was evaluated using tolerance and variance inflation factor. The co-linearity was assessed with and without intercept, and no issues were found.

### 3.3 Viral load kinetics

Viral load kinetics, including maximum viral load, duration of viremia, and individual AUC calculated using the trapezoidal method, were assessed for their diagnostic accuracy in predicting patient death or graft failure. For multiple CMV viremia episodes, cumulative viral load measures were calculated and included cumulative duration, overall maximum viral load, and cumulative individual AUC. Unadjusted and adjusted receiver operating characteristic (ROC) curve models were used to evaluate relationship between cumulative viral load kinetics and outcomes. The ROC AUC was modeled to assess the diagnostic accuracy of the model, and the Youden index was applied to determine the optimal cutoff point for sensitivity and specificity.

### 3.4 Multistate modeling

A 3-state Prentice-Williams-Peterson gap time (time between successive events) survival model was used to analyze the progression of individuals through various states of CMV infection. This model is specifically designed to handle recurrent events in the context of multistate modeling and allows for a detailed examination of the transitions between repeated CMV episodes, accommodating both the timing and sequence of events. It extends the Cox proportional hazards model to accommodate repeated occurrences of events within individuals.

The disease progression is modeled through a set of 3 states with pre-defined states from transplantation (TX) to death-censored graft failure: State 1: TX → first CMV episode, TX → graft failure; State 2: first CMV episode → second CMV episode, first CMV episode → graft failure; State 3: second CMV episode → graft failure.

### 3.5 Survival analysis

Kaplan-Meier analyses with survival probability compared using the logrank test were used to assess the relationship between long-term consequences of CMV infection, including the impact on graft and overall survival. Unadjusted and adjusted hazard ratios (HRs) were calculated with a Cox proportional hazard model. The log(−log[survival]) versus log of survival time and interaction were used to check Cox proportionality assumptions. The average time to event was determined using the mean cumulative function, provided by the cumulative distribution function, which was used to calculate the expected time to failure.

## 4 Results

### 4.1 Clinical cohort and principal events

#### 4.1.1 Patient demographics and outcomes

**Figure 1:**
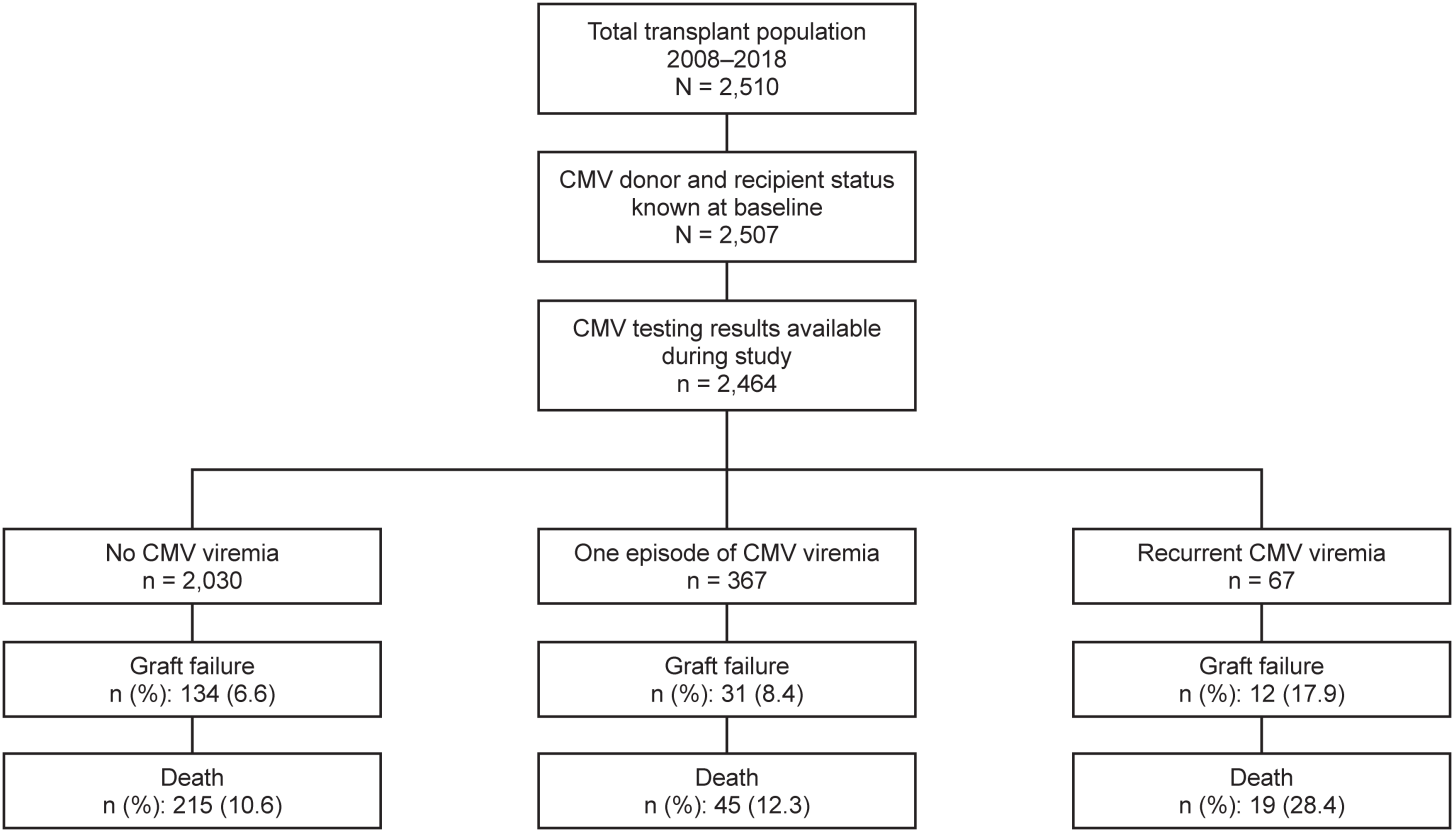
Disposition of patients in the study cohort who received a kidney transplant during the observation period of 2008–2018.

Donor and recipient CMV serostatus and post-transplant CMV viral load measurements were recorded in 2,464 (98.3%) patients (9), who formed the basis of this analysis (**Figure 1**). As shown in **Table 1**, 62.7% were male, the mean age (± standard deviation [SD]) was 51.9 ± 15.2 years, and 3.0% were pediatric patients (<19 years). In all, 1,568 recipients and 1,337 donor organs were CMV seropositive, and 35.8% of donor/recipient pairs were D+/R+, 18.4% were D+/R-, 27.8% were D-/R+, and 17.9% were D-/R-. A total of 1,123 (45.6%) patients received antiviral prophylaxis, principally with valganciclovir or ganciclovir sodium, which continued for a median of 82 (range: 2–1,638) days.

**Table 1:** Patient demographics, primary diagnosis, antiviral prophylaxis, death rate, and graft failure rate by number of CMV viremia episodes.

Number of episodes	0 (n = 2,030)	1 (n = 367)	2+ (n = 67)	*P*-value***
Sex, n (%)				
Male	1,279 (63.0)	224 (61.0)	42 (62.7)	0.7728
Female	751 (37.0)	143 (39.0)	25 (37.3)	
Age <19 years, n (%)*				
Yes	71 (3.5)	3 (0.8)	1 (1.5)	0.7728
No	1,959 (96.5)	364 (99.2)	66 (98.5)	
Age, years*				
Minimum	1.7	6.6	16.6	0.0000
Median	53.2	58.4	60	
Maximum	81.1	82	77.6	
Standard deviation	15.3	14.5	12.3	
Mean	51.1	55.1	58.2	
Mean 95% CI	(50.4, 51.7)	(53.6, 56.6)	(55.2, 61.2)	
Race, n (%)				
Caucasian/White	1,246 (61.4)	193 (52.6)	33 (49.3)	0.0014
Other	784 (38.6)	174 (47.4)	34 (50.8)	
CMV D/R status at baseline, n (%)				
D-/R-	438 (21.6)	2 (0.5)	2 (3.0)	0.0000
D-/R+	600 (29.6)	75 (20.4)	10 (14.9)	
D+/R-	299 (14.7)	125 (34.1)	30 (44.8)	
D+/R+	693 (34.1)	165 (45.0)	25 (37.3)	
Primary diagnosis, n (%)				
Glomerulonephritis	710 (35.0)	98 (26.7)	20 (29.9)	0.0206
Diabetes	450 (22.2)	93 (25.3)	12 (17.9)	
Other	870 (42.9)	176 (48.0)	35 (52.2)	
Prophylaxis, n (%)**				
Yes	829 (40.8)	242 (65.9)	52 (77.6)	0.0000
No	1,201 (59.2)	125 (34.1)	15 (22.4)	
Duration of prophylaxis, days**				
Minimum	2.0	2.0	3.0	0.0560
Median	78.0	94.5	104.0	
Maximum	867.0	596.0	1638.0	
Standard deviation	88.3	98.2	244.4	
Mean	101.4	119.2	154.0	
Mean 95% CI	(95.3, 107.4)	(106.7, 131.6)	(86.0, 222.0)	
Death, n (%)				
Survived	1,815 (89.4)	322 (87.7)	48 (71.6)	0.0000
Died	215 (10.6)	45 (12.3)	19 (28.4)	
Graft failed, n (%)				
No	1,896 (93.4)	336 (91.6)	55 (82.1)	0.0012
Yes	134 (6.6)	31 (8.5)	12 (17.9)	

*Age at first transplantation.

**Prophylaxis refers to any administration of CMV treatment drugs within first 14 days post-transplant.

***P-value for comparison by number of CMV viremia episodes.

CI, confidence interval; CMV, cytomegalovirus; D, donor; R, recipient.

#### 4.1.2 CMV infection

**Figure 2:**
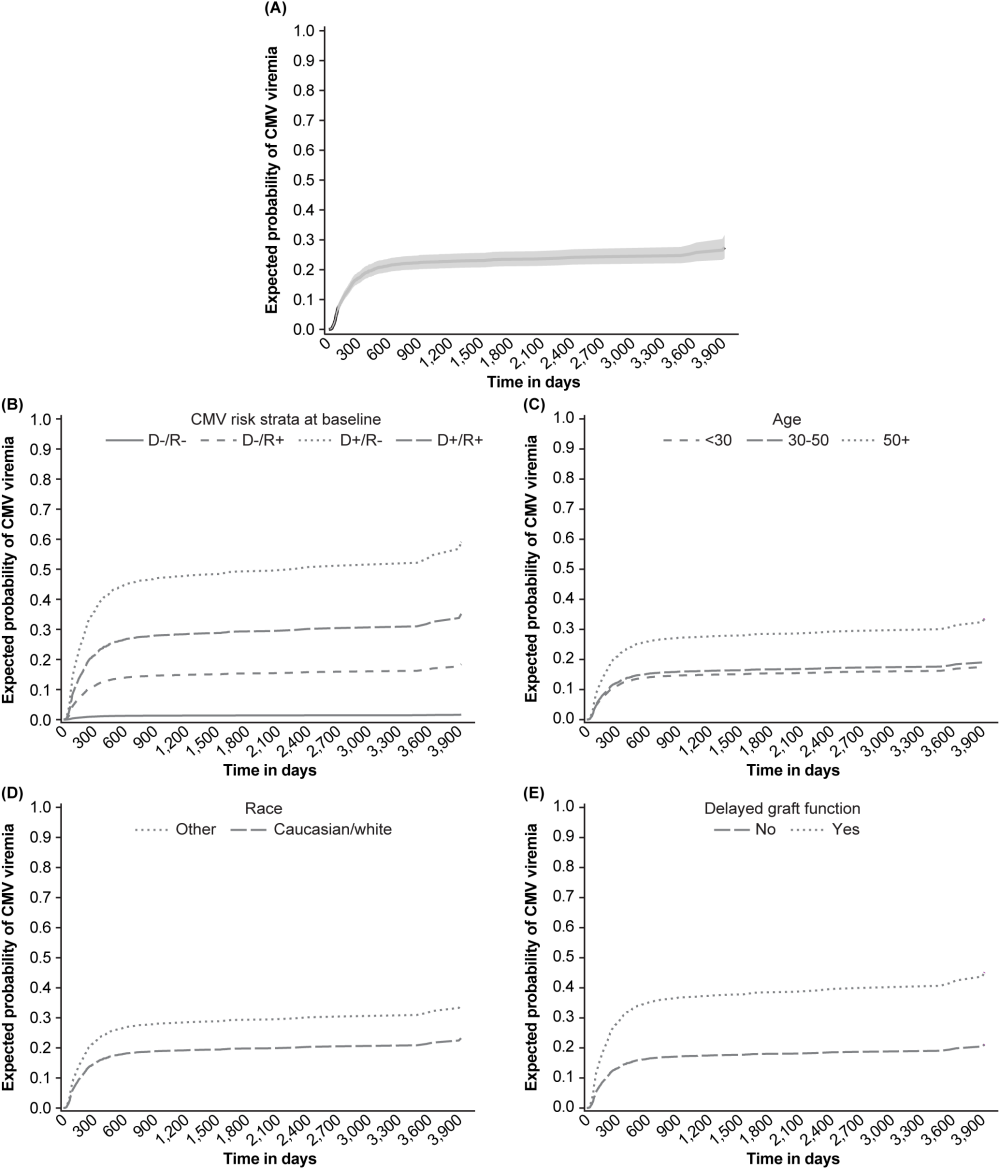
Cumulative probability for multiple cytomegalovirus (CMV) infections. **(A)** Overall, **(B)** donor (D)/recipient (R) CMV status, **(C)** age, **(D)** race, and **(E)** delayed graft function. Panel **(B)** reprinted from Dobrer et al., 222.8: Effect of recurrent CMV viremia on graft and patient survival in renal transplantation, Transplantation, Vol 108, Issue 9S with permission Wolters Kluwer Health. Inc.

CMV infection occurred in 434 patients, of whom 367 (84.6%) had a single episode and 67 (15.4%) had recurrent viremia occurring a mean (± SD) 278 ± 472 days after transplantation. Mean cumulative incidence analysis (**Figure 2**) confirmed that almost all recurrent viremic episodes occurred within the first 18 months post-transplant. Approximately one quarter of these had CMV viremia only, whereas 65.1% had clinical complications and 8.4% were hospitalized. Descriptive analysis (**Table 1**) and adjusted multinomial logistic regression (**Table 2**) showed that increased recipient age (OR: 1.02, *p*<0.0001 vs 1.04, *p*<0.0001), non-Caucasian race (OR: 1.48, *p*=0.0036 vs 2.69, *p*=0.0014), D+/R- CMV serostatus (OR: 2.18, *p*<0.0001 vs 4.29, *p*<0.0002), and delayed graft function (OR: 1.56, *p*<0.0006 vs 3.05, *p*<0.0001) described an important risk phenotype for both first and recurrent CMV infections, respectively. Results from the unadjusted multinomial logistic regression analysis are shown in **Supplementary Table 1**.

**Table 2:** Adjusted multinomial regression analysis examining risk phenotype related to increased risk of first and recurrent CMV infections.

	1 Episode, OR (95% CI)	*P*-value	2+ Episodes, OR (95% CI)	*P*-value
Age*	1.02 (1.01, 1.03)	<0.0001	1.04 (1.02, 1.06)	<0.0001
Race				
Other	1.48 (1.14, 1.93)	0.0036	2.69 (1.47, 4.93)	0.0014
Caucasian/White	Reference			
Primary diagnosis				
Diabetes	0.83 (0.61, 1.11)	0.2072	0.45 (0.23, 0.90)	0.0246
Glomerulonephritis	0.65 (0.49, 0.87)	0.0030	0.69 (0.39, 1.24)	0.2125
Other	Reference			
CMV status at baseline (D/R)				
D+/R-	2.18 (1.50, 3.16)	<0.0001	4.29 (2.00, 9.16)	0.0002
D-/R+	0.50 (0.37, 0.67)	<0.0001	0.41 (0.19, 0.88)	0.0223
D-/R-	0.03 (0.01, 0.11)	<0.0001	0.28 (0.06, 1.27)	0.0983
D+/R+	Reference			
Induction ATG				
Yes	1.39 (0.98, 1.98)	0.0628	1.10 (0.57, 2.14)	0.7757
No	Reference			
Delayed graft function				
Yes	1.56 (1.21, 2.02)	0.0006	3.05 (1.80, 5.18)	<0.0001
No	Reference			
On prophylaxis				
Yes	1.24 (0.85, 1.81)	0.2611	2.05 (0.91, 4.65)	0.0849
No	Reference			

Reference category is No CMV episodes.

*Age at first transplantation.

CI, confidence interval; CMV, cytomegalovirus; D, donor; OR, odds ratio; R, recipient.

#### 4.1.3 Patient and graft outcomes

**Figure 3:**
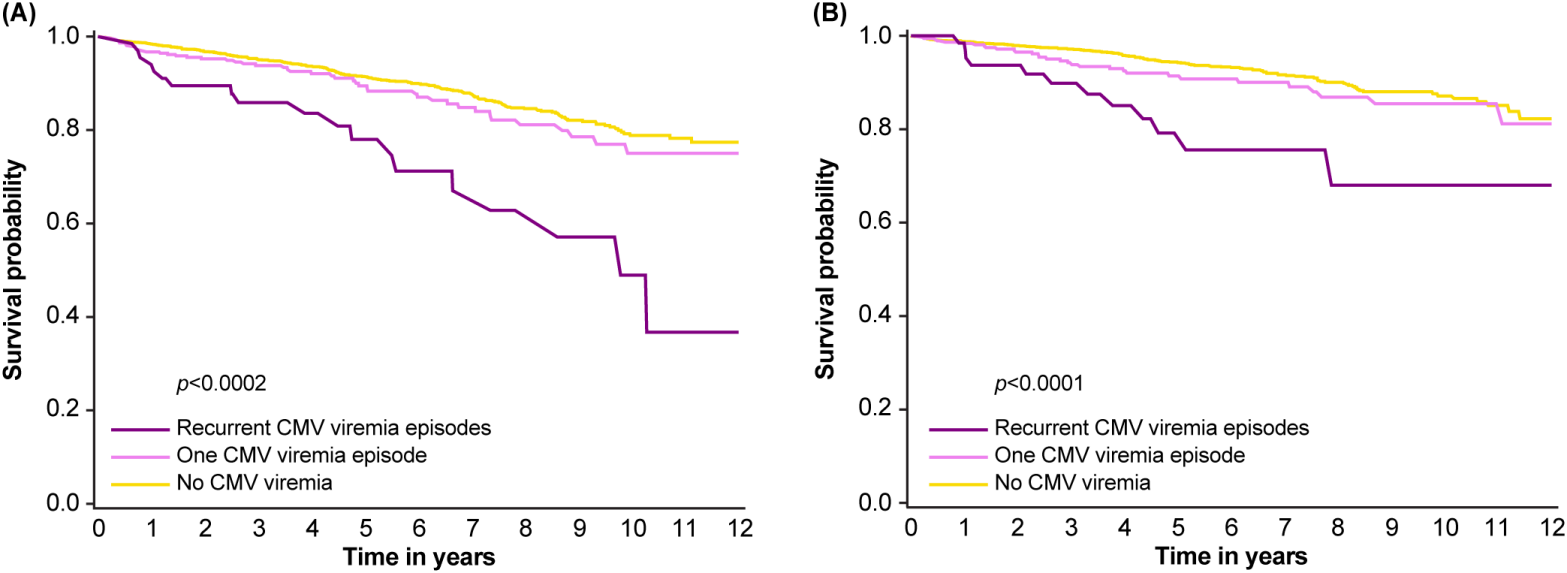
Survival in patients with or without recurrent cytomegalovirus (CMV) infection post-transplant. **(A)** Patient survival and **(B)** death-censored graft survival. Panel **(A)** reprinted from Dobrer et al., 222.8: Effect of recurrent CMV viremia on graft and patient survival in renal transplantation,Transplantation, Vol 108, Issue 9S with permission Wolters Kluwer Health. Inc.

**Figure 4:**
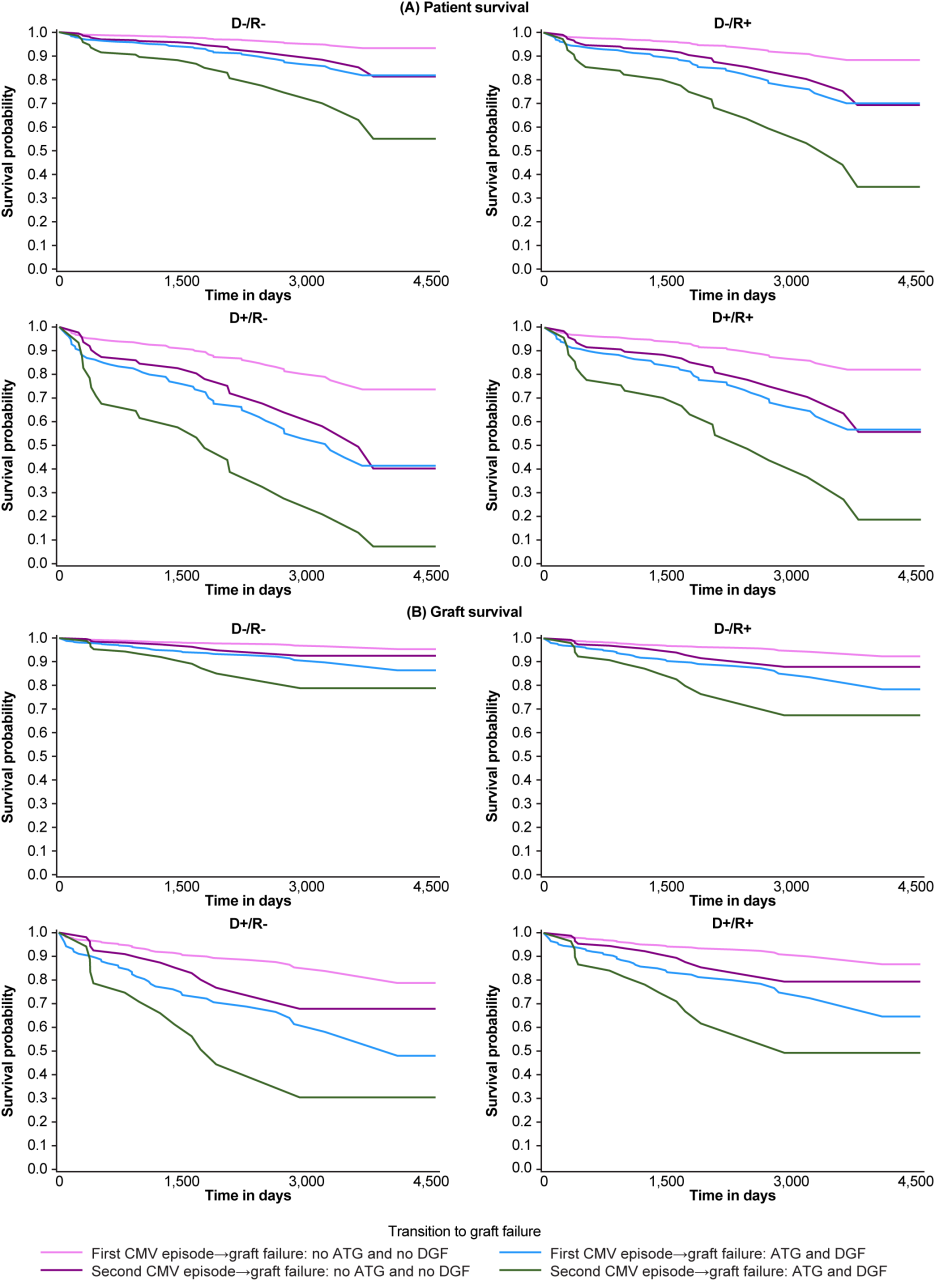
Patient **(A)** and graft **(B)** survival according to primary or recurrent cytomegalovirus (CMV) infection and defined risk categories from a multistate model: State 1: TX to first CMV episode, TX to graft failure; State 2: first CMV episode to second CMV episode, first CMV episode to graft failure; State 3: second CMV episode to graft failure. ATG, anti-thymocyte globulin; D, donor; DGF, delayed graft function; R, recipient; TX, transplantation.

A total of 279 (11.3%) patients died and a further 177 (7.2%) patients lost their graft during the period of follow-up. Kaplan-Meier analysis showed that patient (**Figure 3A**) and graft (**Figure 3B**) survival were comparable in patients who had no viremia or only a single episode of viremia but were both significantly reduced in those with recurrent CMV infection (death-censored graft survival, *p*<0.0001; patient survival, *p*<0.0002). Graft loss increased from 6.6% in the 2,030 patients without CMV infection, to 8.4% in the 367 patients with a single episode of viremia and 17.9% in those with recurrent CMV infection, whereas mortality rose from 10.6% to 12.3% and 28.4%, respectively. Stratified survival analysis from multistate model analysis confirmed that D+/R- graft recipients receiving ATG with DGF were at exceptional risk of graft loss (**Figure 4A**) or death (**Figure 4B**), with patient and graft survival probabilities of less than 30% and 7%, respectively, at 12 years post-transplant.

### 4.2 CMV viral load kinetics

#### 4.2.1 Measures of viremia

**Figure 5:**
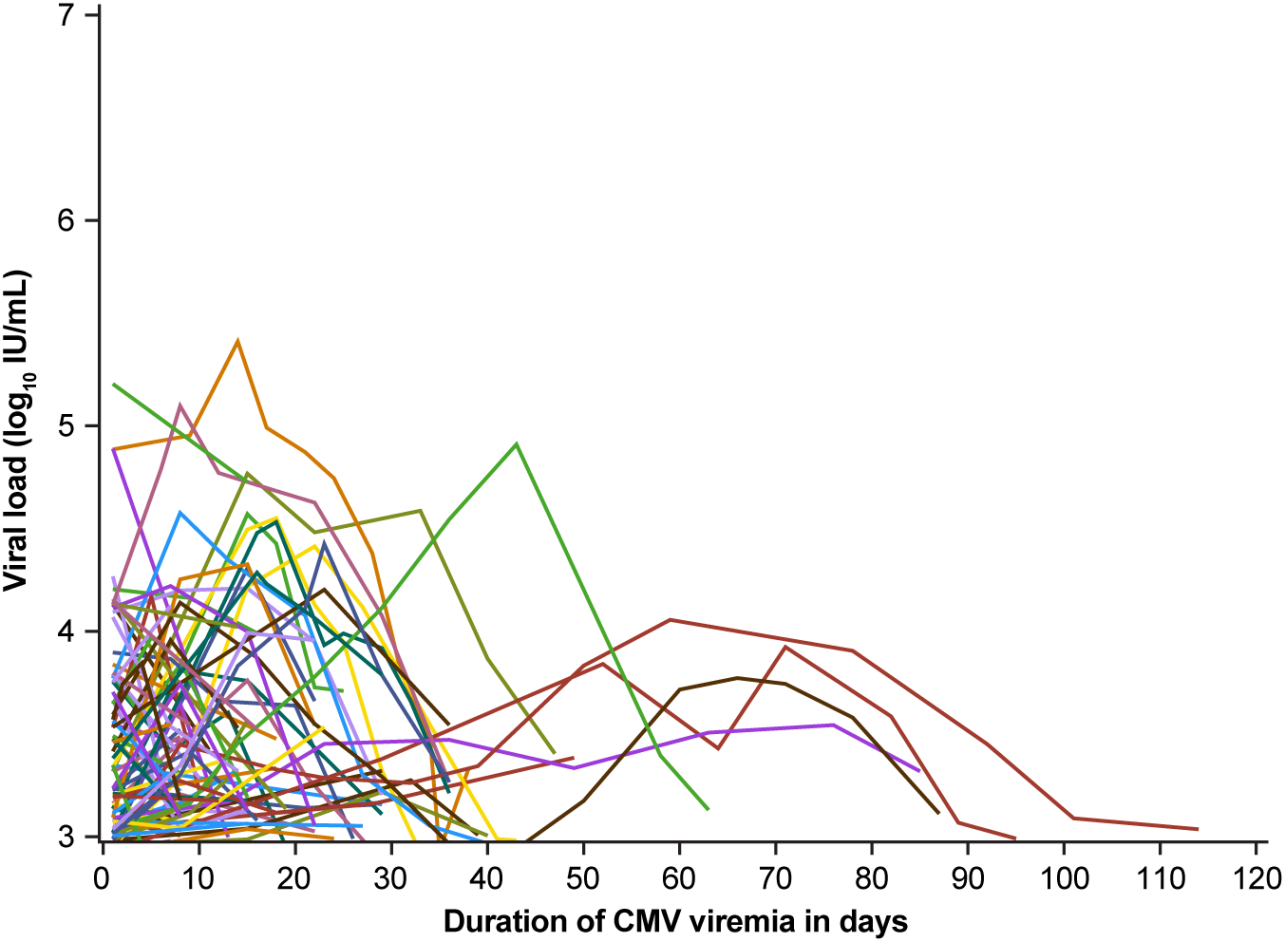
Viral load and duration of viremia in patients with recurrent cytomegalovirus (CMV) infection (n = 67 patients, 150 episodes).

Viral load kinetics were measured as maximum viral load, duration of viremia, and integral (AUC) of viral exposure over time. The heterogeneity of these parameters in the 150 episodes of recurrent CMV viremia is shown in **Figure 5**.

Quantitative viral exposure was comparable in first and recurrent infections as shown in **Table 3**. The mean duration of viremia was 18.2 days for a first infection and 23.4 days for recurrent infections. Mean CMV viral AUC was correspondingly 73.8 log_10_ IU/mL*days for the first episode and 89.8 log_10_ IU/mL*days for recurrent infections. Measures of viral exposure also increased slightly, although not significantly, according to clinical severity. Mean duration of viremia was 15.8 days in patients with a first episode of viremia alone and 23.5 days in those hospitalized with clinical complications, compared with 13.6 days and 33.1 days, respectively, among patients with a recurrent infection. Mean AUC was 57.4 log_10_ IU/mL*days in first episodes with CMV viremia only, compared with 107.5 log_10_ IU/mL*days in those with clinical complications and hospitalization. The corresponding values were 47.9 log_10_ IU/mL*days and 147.2 log_10_ IU/mL*days among those with recurrent infection (*p*<0.0004).

**Table 3:** Summary of CMV viral load kinetics by episode.

CMV episode	1 Episode	2+ Episodes	Total	*P*-value
*Duration of episode, mean (SD)*	*18.2 (10.8)*	*23.4 (20.3)*	*19.0 (12.9)*	*0.2548*
CMV viremia only	15.8 (7.2)	13.6 (6.2)	15.4 (7.1)	0.196
CMV viremia with clinical complications	18.6 (10.9)	26.1 (23.5)	19.6 (13.7)	0.150
CMV viremia with clinical complications and hospitalization	23.5 (18.4)	33.1 (9.7)	25.9 (17.0)	0.056
*Maximum viral load (log_10_), mean (SD)*	*4.0 (0.7)*	*3.8 (0.6)*	*4.0 (0.7)*	*0.0721*
CMV viremia only	8.5 (1.2)	8.2 (1.4)	8.4 (1.2)	0.161
CMV viremia with clinical complications	9.3 (1.7)	8.7 (1.2)	9.2 (1.7)	0.061
CMV viremia with clinical complications and hospitalization	10.6 (2.3)	10.3 (0.8)	10.5 (2.0)	0.577
AUC (log_10_ IU/mL*days), mean (SD)	*73.8 (53.2)*	*89.8 (81.1)*	*76.4 (58.8)*	*0.5481*
CMV viremia only	57.4 (29.5)	47.9 (23.4)	55.6 (28.6)	0.1629
CMV viremia with clinical complications	76.4 (54.6)	99.4 (92.1)	79.7 (61.8)	0.3862
CMV viremia with clinical complications and hospitalization	107.5 (84.4)	147.2 (44.9)	117.4 (77.7)	0.0824

AUC, area under the curve; CMV, cytomegalovirus; SD, standard deviation.

**Figure 6:**
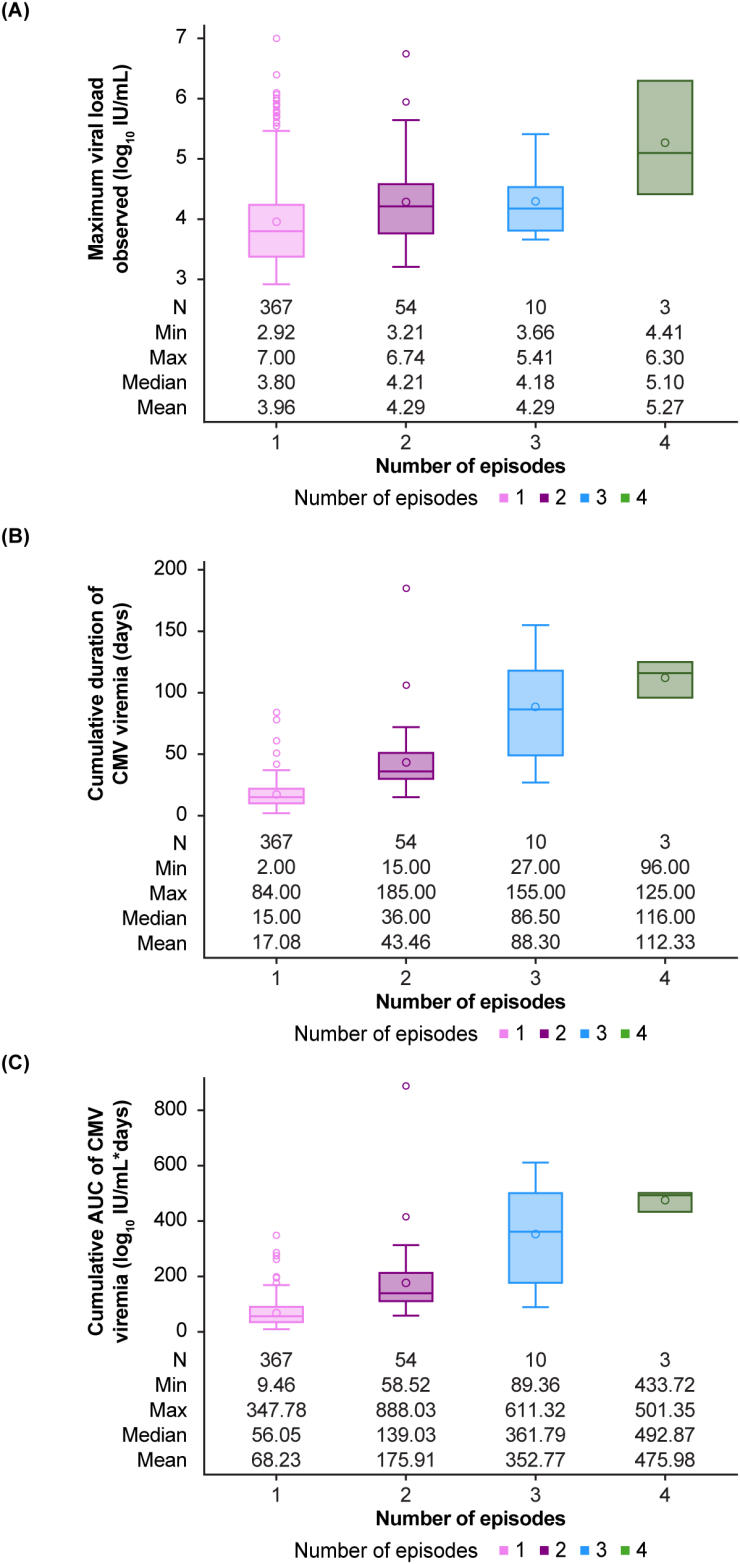
Cumulative values of viral load kinetics combining all cytomegalovirus (CMV) viremia episodes per patient. **(A)** Maximum viral load, **(B)** duration of viremia, **(C)** individual area under the curve (AUC).

Cumulative viral load kinetics (i.e., combining first and recurrent episodes of CMV infection) are shown in **Figure 6**. The median maximum viral load (**Figure 6A**) was 3.8 log_10_ IU/mL for patients with a single episode of viremia, rising to 4.2 log_10_ IU/mL for those with 2 viremic episodes, then to 4.2 log_10_ IU/mL for those with 3 episodes, and finally to 5.1 log_10_ IU/mL in patients with 4 episodes of viremia. The median duration of viremia (**Figure 6B**) was 15 days for patients with a single episode of viremia; this increased almost 3-fold to a median of 36 days for those with 2 viremic episodes, then 5-fold to a median of 86.5 days for those with 3 episodes and over 7-fold to a median of 116 days for the patients with 4 episodes of viremia. The cumulative viral AUC (**Figure 6C**) rose in parallel, from a median of 56.1 log_10_ IU/mL*days in those with 1 episode of viremia to 139 log_10_ IU/mL*days in those with 2 episodes, to over 6-fold to 361.8 log_10_ IU/mL*days in patients with 3 episodes and over 8-fold to 492.9 log_10_ IU/mL*days in those with 4 episodes of viremia.

**Figure 7:**
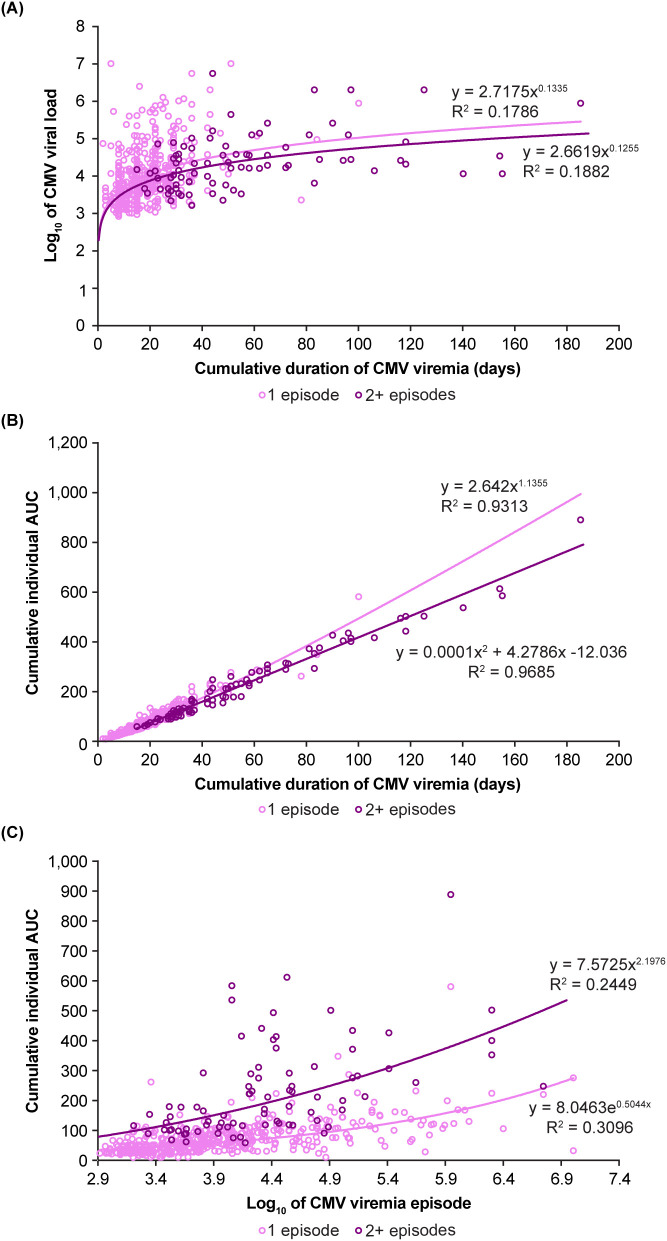
Relationship between individual measures of cumulative viral load kinetics. The cumulative duration of viral infection is significantly related to the cumulative area under the curve (AUC), whereas maximum viral load is less strongly related to the AUC. **(A)** Shows maximum viral load and duration of viremia, **(B)** shows viral load AUC and duration of viremia, **(C)** shows viral load AUC and maximum viral load. CMV, cytomegalovirus.

**Figure 7** shows the significant correlation between cumulative CMV AUC and cumulative duration of CMV viremia (*p*<0.0001). As anticipated, the relationship between maximum viral load and cumulative AUC (**Figure 7C**) was less close when analyzed across all episodes, reflecting the fact that a single maximum measure taken from repeated episodes of infection is less deterministic of overall viremic exposure.

#### 4.2.2 Influence of CMV viral load

**Figure 8:**
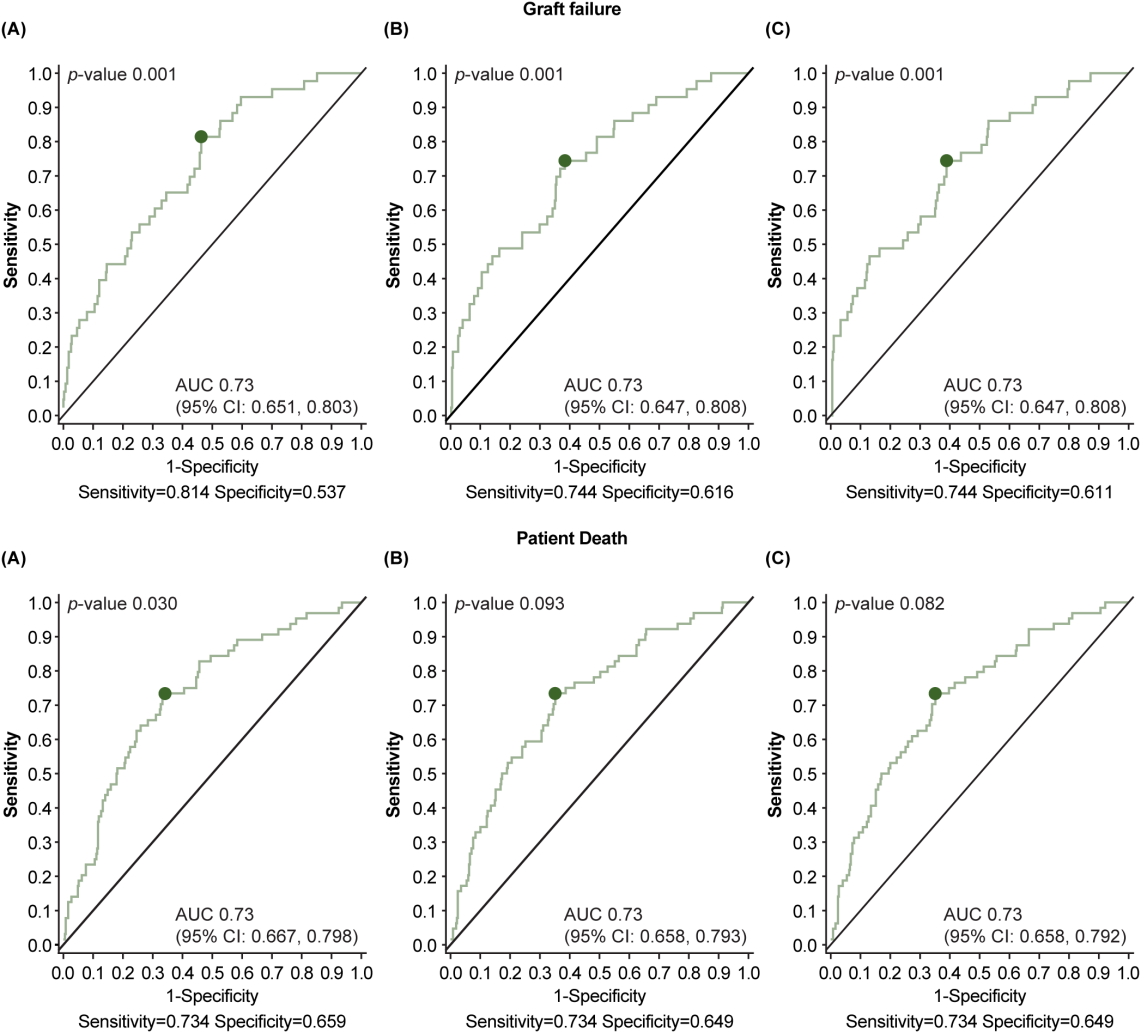
Receiver operator characteristic (ROC) analysis for cytomegalovirus (CMV) viral load kinetics. Figures show ROC area under the curve (AUC) analysis of: **(A)** maximum viral load, **(B)** duration of viremia in days, and **(C)** viral load over time. Numbers given in parentheses are adjusted ROC AUC. Top row shows graft failure, bottom shows patient death. Covariates for each adjusted model were: CMV donor/recipient status at baseline, ATG induction, DGF, race, sex, age, and antiviral prophylaxis. ATG, anti-thymocyte globulin; DGF, delayed graft function.

ROC curves were constructed to examine the quantitative relationship between maximum viral load (**Figure 8A**), duration of viremia (**Figure 8B**) or viral load over time as measured by AUC (**Figure 8C**), and transplant failure or death. For graft failure, adjusted ROC analysis of CMV maximum viral load provided an AUC of 0.73 (*p*<0.001); for duration of viremia, the AUC was 0.73 (*p*<0.001); and for viral load over time, the AUC was 0.73 (*p*<0.001). For patient death, adjusted ROC analysis of CMV maximum viral load provided an AUC of 0.73 (*p*<0.03); for duration of viremia, the AUC was 0.73 (*p*<0.09); and for viral load over time, the AUC was 0.73 (*p*<0.08).

**Figure 9:**
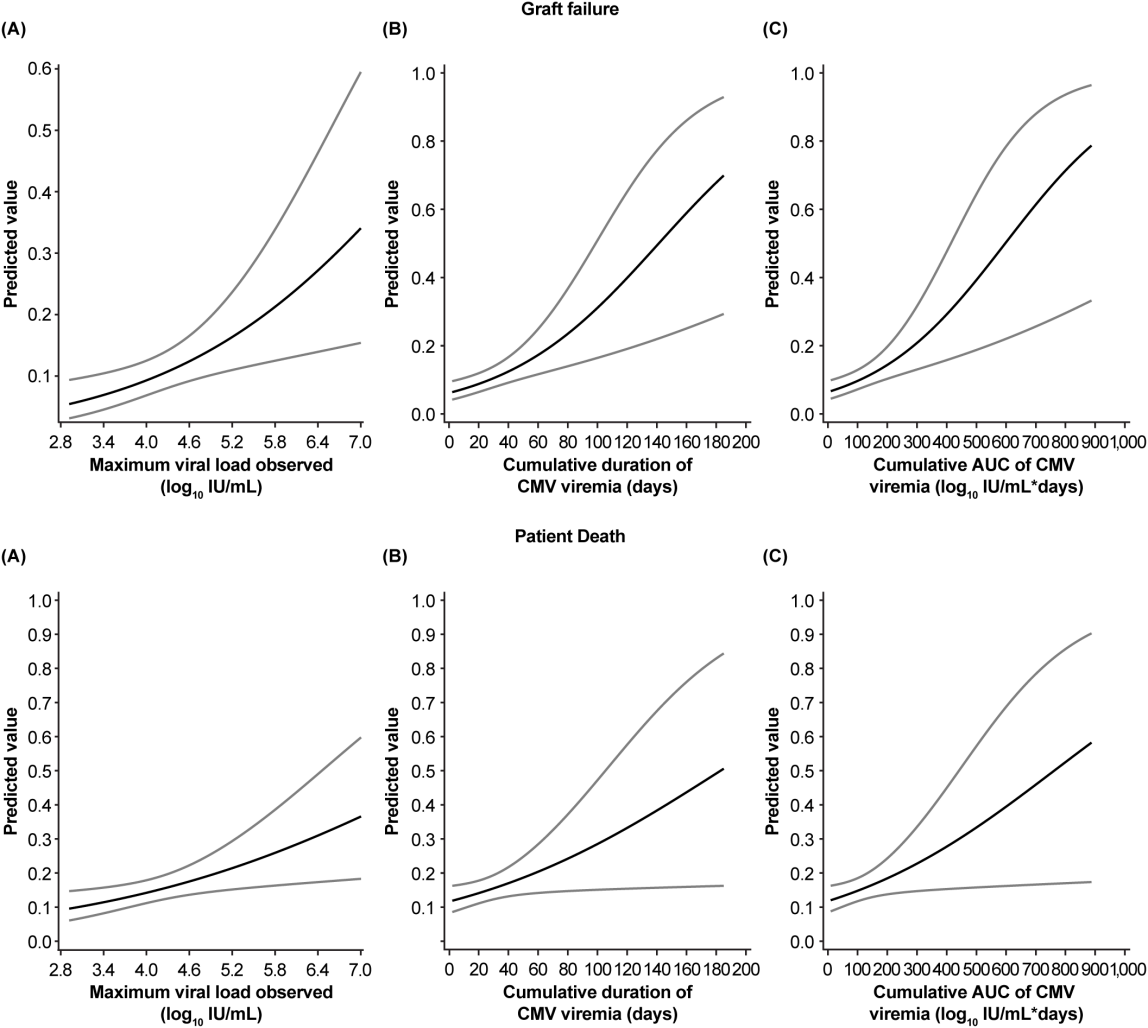
Relationship between death-censored graft failure and patient death and cumulative cytomegalovirus (CMV) viral load kinetics. Figures show predicted graft failure and patient death according to **(A)** maximum CMV viral load in log_10_ IU/mL, **(B)** duration of viremia in days, and **(C)** viral area under the curve (AUC) in log_10_ IU/mL*days. Top row shows graft failure, bottom shows patient death. Light grey lines represent 95% confidence intervals.

As shown in **Figure 9**, the predicted probability of graft failure was closely related to both the duration of viremia (**Figure 9B**) and total viral load as measured by AUC (**Figure 9C**), increasing from approximately 5%–80% across the range of 0–190 days and 0–900 log_10_ IU/mL*days. The probability of patient death behaved similarly, rising from 0 to over 50% across the same value ranges. In both cases, the probability of failure was less closely related to maximum viral measurement (**Figure 9A**), consistent with the observation that there was only a minimal increase in this value during recurrent CMV infections.

## 5 Discussion

Accurate and uniform monitoring of viral load (12) and rigorous use of antiviral therapy have mitigated, although not eliminated, the devastating consequences of CMV infection and modulated the important early indirect consequences and costs of care. Within this framework, our prior and current reports from a large longitudinal study of real-world evidence in current practice explore the characteristics and kinetics of primary and recurrent CMV viremia and document their impact on the success of kidney transplantation. Findings from the current analysis highlight the striking incremental risk of recurrent CMV infection, which substantially increases the probability of both premature graft loss and patient death. The analysis identified quantitative viremia, as measured by viral load kinetics, as a key explanatory factor, emphasizing that increased vigilance and more effective therapy are urgently required in this high-risk group.

Despite rigorous application of treatment guidelines with standardized use of antiviral prophylaxis, viral monitoring and preemptive therapy, 18% of transplanted patients developed CMV infection and, of those, 15% had recurrent viremia. The majority of all infectious episodes occurred during the first 18 months post-transplant, defining the period of maximum risk and required vigilance. Demographic characteristics were similar for patients with both first and recurrent infections, who were predominantly older, non-Caucasian recipients of a D+/R- graft who experienced delayed graft function (DGF) post-transplant, defining a high-risk profile (1, 13). Clinical expression was similar for both primary and recurrent episodes; approximately one quarter of cases presented as asymptomatic viremia, three quarters as CMV disease with hematological, gastrointestinal, hepatic, pulmonary, ocular, and other consequences, and 5% were hospitalized with CMV disease (1, 14). Transplant success was markedly reduced in patients with recurrent CMV infection: both graft and patient survival declined rapidly throughout the first 6 years, at which time over 70% of grafts in the highest risk group (D+/R-) had been lost.

We have shown previously that viral load kinetics serve as important predictors of premature transplant failure in patients with a first episode of CMV infection (9). These measurements provided a simple heuristic in which a maximum viral load >10,000 IU/mL, duration of viremia >15 days, or an AUC of >60 log_10_ IU/mL*days during a first episode of viremia provide a valuable tool to inform early and active therapy and provide a potential surrogate marker in clinical trial settings (9). We extend and refine the relevance and importance of these measurements in this second article, providing a simple sequential heuristic pathway to determine incremental risk. The composite risk phenotype of D+/R- status, increased age, non-Caucasian race, ATG use, and DGF identifies patients at elevated risk within the first week post-transplant, permitting meticulous oversight throughout the transplantation course. This hazard rises dramatically in patients who have recurrent episodes of CMV viremia, and particularly in those with a cumulative duration of over 60 days of CMV viremia or a cumulative AUC of over 300 log_10_ IU/mL*days, both of which are highly predictive of transplant failure measured by graft loss or patient death. This highlights the importance of identifying patients with recurrent CMV viremia, or prolonged viremia over 15 days, especially in the presence of composite risks such as DGF. These patients can then be reviewed closely to consider adjustment in antiviral therapy or immunosuppressive therapy according to CMV genotype, therapeutic resistance, leukopenia, and other factors associated with adverse outcomes. These simple strategies can be quickly and easily adopted into clinical practice to highlight patients at risk.

Optimizing treatment of these patients, however, remains challenging. Systematic review of 41 recent trials with over 5,000 graft recipients provides nuanced information (15). Although there is high-certainty evidence to support the use of antiviral prophylaxis, there is only moderate certainty to confirm that this reduces the risk of death from CMV disease, or to specify the current ideal agent, duration of therapy, or optimal dose to maximize therapeutic benefit (15). However, this analysis confirms with moderate certainty that extending the duration of prophylaxis beyond the period of 3–6 months, which is common practice in Canadian and other centers, or perhaps to a point at which external immune suppression has been reduced, might be more effective in reducing the risk of CMV (15). Furthermore, results suggest with lower certainty that a reduced dose of 450 mg/day valganciclovir might be equally effective as higher-dose therapy, offering the possibility to reduce neutropenia or graft injury, which may further mitigate CMV risk (16). However, this may entail potential negative consequences such as increased selection for CMV antiviral resistance or lower patient adherence. An alternative approach could be to use a therapy without treatment-limiting neutropenia to support improved patient outcomes.

The recent approval of 2 new antiviral therapies, letermovir for prophylaxis and maribavir for treatment, further expands the opportunities for care (17–19). Letermovir is a first-in-class antiviral agent with a unique mechanism of action as an inhibitor of the CMV DNA terminase complex, which has been approved for prophylaxis of CMV infection and disease. Large-scale randomized trials confirm that it is non-inferior to valganciclovir, with approximately 10% of patients developing CMV infection in both arms, while causing fewer drug-related adverse events (20% vs 35%) and leukopenia (4% vs 19%) (20). However, letermovir has certain therapeutic challenges, ranging from a lower barrier to resistance, limiting efficacy as an antiviral treatment, to important drug interactions through cytochrome P450 3A4 and other axes, requiring dose modification of key immune suppressants (17). Maribavir, a unique competitive inhibitor of adenosine triphosphate binding to the pUL97 viral protein kinase, offers important advantages for treatment of CMV infection (21, 22), including refractory infection (17, 19, 23, 24), without treatment-limiting neutropenia or nephrotoxicity. Large-scale randomized trials show greater efficacy with maribavir compared with investigator-assigned therapy (valganciclovir/ganciclovir, foscarnet, or cidofovir) (56% vs 24%), with less graft injury (8% vs 21%) and neutropenia (9% vs 34%), along with no increase in treatment-emergent adverse events (23). These agents offer the enticing potential to revise prophylaxis and treatment care that may transform the burden of this virus and further improve patient outcomes.

We have previously indicated a number of limitations associated with this study, including selection bias, information bias, and confounding, which are inherent to observational design (25). To minimize selection bias, the study included all sequential patients who underwent transplantation in a single program in Canada who were followed throughout the period of observation within an integrated care network. Although information bias may occur from many sources, stringent efforts were made to reduce this, using a single provincial electronic database with standard entry practices and uniform analytical strategies. Risk strata and classification criteria were defined according to national and international norms, and the period of enrollment and observation was chosen to ensure standardized diagnostic and therapeutic practices. Although confounding is perhaps more difficult to eliminate, we have made stringent efforts to minimize confounding by indication or by patient risk through *post hoc* stratification and regression modeling. Although the potential for time-varying differences in patient referral, case mix, unit services, and care patterns remain, these reflect normal practice patterns over this period. Finally, as this is a single-center study, the results require confirmation in other settings, where antiviral prophylaxis and induction therapy may vary.

Despite the limitations inherent in observational design, this large, longitudinal study has important strengths, including sample size and provincial scope, the inclusion of sequential patients who underwent transplantation within a standardized care program, and long-term follow-up and management within a uniform clinical and laboratory program. It confirms the serious consequences of CMV infection, which not only causes systemic illness but also triggers inflammatory injury of specific target organs, complicates effective immunosuppression, destabilizes host immunological quiescence, and jeopardizes both graft and patient survival (26–28). The relationship between CMV infection and transplant failure may be causal, related to direct systemic endothelial injury, to the immune-modulating effect of the virus in enhancing targeted T-cell rejection, or to iatrogenic reduction in immune suppression secondary to leukopenia, all leading to progressive vascular destruction, or may be consequential, whereby treatment of rejection increases the risk of viremia (29). We cannot decipher all these interactions at present, which are now the focus of a deeper evaluation. However, the data reported here underscore the adverse consequences of the virus and demonstrate that the simple application of standardized clinical guidelines does not prevent the ravages of this infection. We showed that CMV viral load kinetics are important in predicting outcome and provide a simple pragmatic set of predictor values that may be critical in guiding therapy and may serve as important virological endpoints for therapeutic trials in this disease.

## Data Availability

The datasets presented in this article are not readily available because they are confidential health services data from British Columbia. Requests to access the datasets should be directed to PK on behalf of the British Columbia Transplant Organization.
