# Supplementary material for: Impact of viral load kinetics and recurrent cytomegalovirus infection in kidney transplantation
Source: Front Immunol. 2025 Aug 8;16:1600289. doi: 10.3389/fimmu.2025.1600289 (PMC12371426; doi:10.3389/fimmu.2025.1600289)
Supplement: Supplementary file 1 [file Table1.docx]

**SUPPLEMENTAL TABLE 1 Unadjusted multinomial regression analysis examining risk phenotype related to
 increased risk of first and recurrent CMV infections.**

|  | 1 Episode  OR (95% CI) | *P*-value | 2+ Episodes  OR (95% CI) | *P*-value |
| --- | --- | --- | --- | --- |
| ***Sex*** |  |  |  |  |
| Female | 1.09 (0.87, 1.37) | 0.4728 | 1.01 (0.61, 1.68) | 0.9577 |
| Male |  | Reference |  |  |
| ***Age**** | 1.02 (1.01, 1.03) | <0.0001 | 1.04 (1.02, 1.06) | 0.00 |
| ***Race*** |  |  |  |  |
| Other | 1.43 (1.15, 1.79) | 0.0016 | 1.64 (1.01, 2.67) | 0.0472 |
| Caucasian/White |  | Reference |  |  |
| ***Primary diagnosis*** |  |  |  |  |
| Diabetes | 1.02 (0.78, 1.35) | 0.8793 | 0.66 (0.34, 1.29) | 0.2260 |
| Glomerulonephritis | 0.68 (0.52, 0.89) | 0.0049 | 0.70 (0.40, 1.22) | 0.2107 |
| Other |  | Reference |  |  |
| ***CMV status at baseline (D/R)*** |  |  |  |  |
| D+/R- | 1.76 (1.34, 2.30) | <0.0001 | 2.78 (1.61, 4.81) | 0.0003 |
| D-/R+ | 0.53 (0.39, 0.70) | <0.0001 | 0.46 (0.22, 0.97) | 0.0412 |
| D-/R- | 0.02 (0.01, 0.08) | <0.0001 | 0.13 (0.03, 0.54) | 0.0051 |
| D+/R+ |  | Reference |  |  |
| ***Type of donor*** |  |  |  |  |
| Deceased donor | 1.63 (1.29, 2.06) | <0.0001 | 2.85 (1.59, 5.09) | 0.0004 |
| Living donor |  | Reference |  |  |
| ***Previous transplants*** |  |  |  |  |
| 1+ | 0.72 (0.45, 1.16) | 0.1778 | 0.56 (0.17. 1.80) | 0.3299 |
| 0 |  | Reference |  |  |
| ***Induction ATG*** |  |  |  |  |
| Yes | 1.77 (1.39, 2.24) | <0.0001 | 2.12 (1.29, 3.49) | 0.0031 |
| No |  | Reference |  |  |
| ***Delayed graft function*** |  |  |  |  |
| Yes | 1.76 (1.40, 2.22) | <0.0001 | 3.40 (2.08, 5.56) | <0.0001 |
| No |  | Reference |  |  |
| ***On prophylaxis*** |  |  |  |  |
| Yes | 2.81 (2.22, 3.54) | <0.0001 | 5.02 (2.81, 8.97) | <0.0001 |
| No |  | Reference |  |  |
| ***Start of prophylaxis*** |  |  |  |  |
| On day 2 | 2.86 (2.25, 3.63) | <0.0001 | 5.29 (2.94, 9.52) | <0.0001 |
| On day 3–14 | 2.43 (1.52, 3.87) | 0.0002 | 3.11 (1.01, 9.53) | 0.0475 |
| No |  | Reference |  |  |

Reference category is No CMV episodes.

*Age at first transplantation.

CI, confidence interval; CMV, cytomegalovirus; D, donor; OR, odds ratio; R, recipient.
